# Coral Larvae Move toward Reef Sounds

**DOI:** 10.1371/journal.pone.0010660

**Published:** 2010-05-14

**Authors:** Mark J. A. Vermeij, Kristen L. Marhaver, Chantal M. Huijbers, Ivan Nagelkerken, Stephen D. Simpson

**Affiliations:** 1 Carmabi Foundation, Willemstad, Curaçao, The Netherlands Antilles; 2 Aquatic Microbiology, Institute for Biodiversity and Ecosystem Dynamics, University of Amsterdam, Amsterdam, The Netherlands; 3 Marine Biology Research Division, Scripps Institution of Oceanography, University of California San Diego, La Jolla, California, United States of America; 4 Department of Animal Ecology and Ecophysiology, Radboud University Nijmegen, Nijmegen, The Netherlands; 5 School of Biological Sciences, University of Bristol, Woodland Road, Bristol, United Kingdom; Northeastern University, United States of America

## Abstract

Free-swimming larvae of tropical corals go through a critical life-phase when they return from the open ocean to select a suitable settlement substrate. During the planktonic phase of their life cycle, the behaviours of small coral larvae (<1 mm) that influence settlement success are difficult to observe *in situ* and are therefore largely unknown. Here, we show that coral larvae respond to acoustic cues that may facilitate detection of habitat from large distances and from upcurrent of preferred settlement locations. Using *in situ* choice chambers, we found that settling coral larvae were attracted to reef sounds, produced mainly by fish and crustaceans, which we broadcast underwater using loudspeakers. Our discovery that coral larvae can detect and respond to sound is the first description of an auditory response in the invertebrate phylum Cnidaria, which includes jellyfish, anemones, and hydroids as well as corals. If, like settlement-stage reef fish and crustaceans, coral larvae use reef noise as a cue for orientation, the alleviation of noise pollution in the marine environment may gain further urgency.

## Introduction

Most nearshore site-attached marine organisms complete an early larval stage in the open ocean before settling to benthic habitats. Chemical compounds produced by reef organisms provide important settlement cues for coral larvae [1], [2], but can only be detected when larvae come into close proximity with organisms producing these compounds. Because waterborne compounds can only be detected downcurrent of their source, planktonic coral larvae which are unable to swim against prevailing currents would be unable to use these cues to orient towards preferred settlement locations [3]. Recent work shows that the larvae of fish and decapods can use sound propagating from nearshore marine communities as an orientation cue to guide their return from the open ocean towards suitable habitats for settlement and growth [4]–[7]. The larvae of marine fishes have specialized anatomical features for detecting sound, but, with the exception of some arthropods, these are not present in invertebrates. Some terrestrial invertebrates, however, can use exterior cilia to register and respond to sound waves [8], [9]. Because the larvae of corals are densely covered with exterior cilia, we hypothesized that they may be able to sense and react to underwater sound fields. Sound propagates much further than light underwater both as particle motion [10] and acoustic pressure [11]; the distance depends on frequency and source power, and thus can provide a useful cue for detection of, and orientation towards, suitable settlement habitat. To test this hypothesis, we studied the movement of coral larvae in choice chambers oriented towards underwater speakers playing reef sounds, which consisted of fish calls and grunts and the continuous crackling sound of snapping shrimps [7], [12].

## Results and Discussion

**Figure 1:**
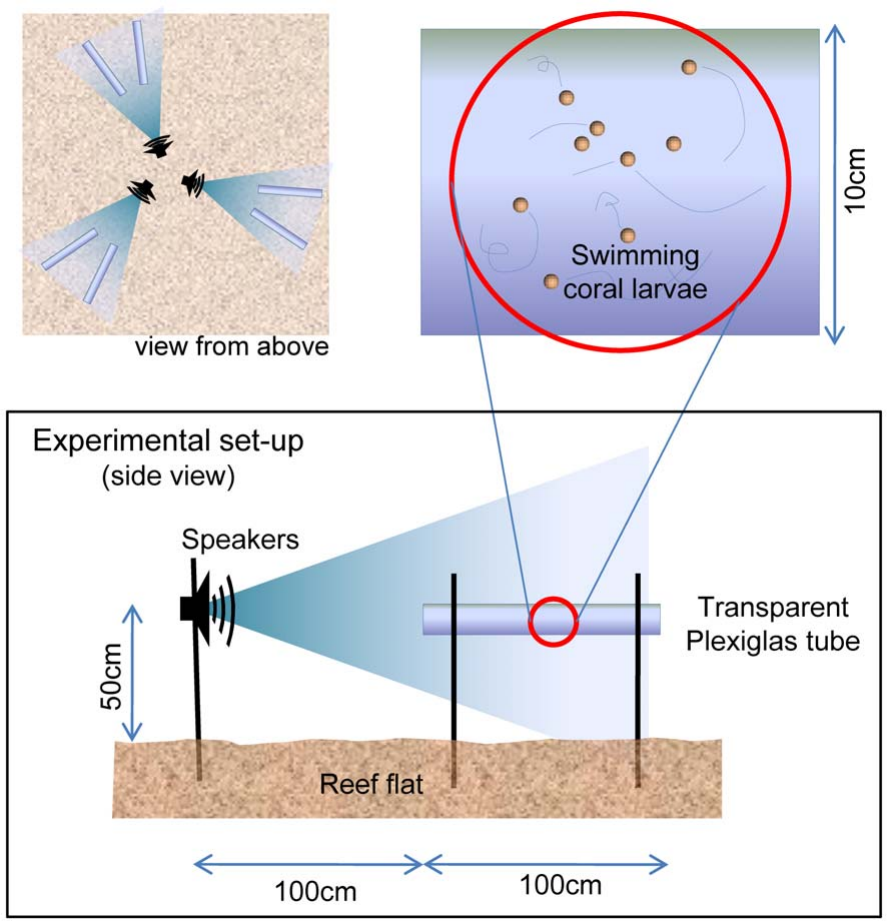
Overview of the experimental setup. The position of coral larvae was observed in six Plexiglas tubes that were arranged around three central underwater loudspeakers to control for the effect of other factors that might influence the movement of larvae (e.g., currents, underwater light fields). Coral larvae are not drawn to scale.

**Figure 2:**
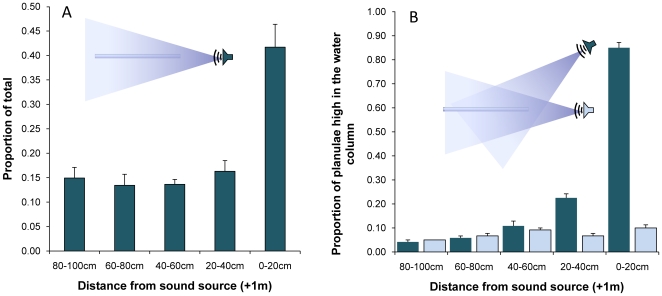
Movement of coral larvae towards reef sounds. (A) The proportion of coral larvae at various distances from speakers playing reef sounds are given as averages of Day 1 and 2 of the experiment (+1SEM). (B) Proportion of larvae at each distance class that were observed against the upper surface of the chambers (i.e., the surface nearest the speakers) when reef sounds were played from above (blue) and sounds were played from aside (light blue). Data are shown as averages from Day 3 of the experiment (+1SEM).

In each trial, using six chambers directed towards underwater speakers playing a compilation of day and night reef sounds (Figure 1), free-swimming coral larvae moved predominantly towards the speakers independent of chamber orientation (Figure 2A, χ^2^ = 30.50, df = 4, p<0.0001). When the chambers were placed 0.5 m below the speakers, larvae moved towards the upper surface of the chambers (i.e., the surface nearest to the speakers) (Figure 2B, F_4,25_ = 431.8, p<0.0001). When the speakers were silent, larvae distributed themselves randomly throughout the chambers independent of the loudspeakers' position (χ^2^ = 0.05, df = 4, p = 0.97). In sum, coral larvae displayed directional movement both horizontally and vertically towards underwater speakers broadcasting reef noise.

The possibility that the directional movement of larvae was caused by moonlight, tides, or chemical cues with onshore-offshore gradients was eliminated by the radial arrangement of the speakers and chambers (Figure 1). Regardless of the orientation of the chamber relative to the shore, larvae in each of the six chambers consistently moved towards the speakers. Movement towards the source of the reef sounds indicates that coral larvae are capable of detecting and responding to acoustic cues in a directional manner. Each chamber was levelled underwater to eliminate directional movement due to depth. The upward vertical movement of larvae towards the source when speakers were positioned higher than the chambers is particularly interesting as it reveals that in our experimental setup planulae showed a preference for sound that overrides the tendency for competent coral larvae to swim down towards the reef substrate [13]. In a field situation except for sounds from mobile soniferous fishes, most reef sounds will propagate upwards from the benthos with some intrinsic directionality [4] thus providing a cue for coral larvae in the overlying water column to move downwards to the reef.

This study reports the first known behavioural response to a water-bourne acoustic cue in a marine larva of the invertebrate phylum Cnidaria, which in addition to jellyfish, anemones and hydroids, includes the corals responsible for the formation of the largest biological structures on earth: coral reefs. Other major sensory modalities which enable detection of light (photoreception), substrates (mechanoreception) and chemicals (chemoreception) have all previously been demonstrated in coral larvae (e.g. [5], [13], [14]). The extent to which an acoustic response facilitates orientation and movement of coral larvae towards suitable settlement habitats is unknown, and will depend on the exact mechanism by which coral larvae detect and respond to sound. In fishes, there is a clear difference in the range of detection between generalists which detect only the particle motion component of acoustic cues (in part by external neuromasts in the lateral line similar to the cilia of coral planulae), and specialists which can also detect acoustic pressure (through anatomical linkages between the gas-filled swimbladder and the otoliths) [4]. We anticipate that coral larvae respond to particle motion, which depending on their sensitivity will limit the likely distances of detection to 10 s to 100 s metres [15]. The fact that coral larvae respond to sound has important implications for understanding dispersal and recruitment success, and warns against treating larvae as passive particles in connectivity models that predict dispersal based on ocean currents alone (for a recent discussion of this issue, see: [16]). Because biological sounds produced by reef organisms propagate metres to kilometres away from reefs [11], their role as a beacon for pelagic life stages of marine invertebrates deserves critical attention, especially because settlement habitat is patchy and often rare in large open bodies of water. If reef sounds provide an orientation cue for free-swimming coral larvae, as they do for settlement-stage coral reef fish larvae and crustaceans [4]–[7], the alleviation of noise pollution in marine environments may gain further urgency and represent yet another factor threatening coral reefs around the world.

## Materials and Methods

### Ethics statement

All animal manipulations were approved by the Department of Environment & Nature (MINA) of the government of the Netherlands Antilles.

### Experimental design

We reared swimming larvae of the dominant Caribbean reef building coral *Montastraea faveolata* during the 2008 mass spawning in Curaçao, Netherlands Antilles. Larvae were raised from gamete bundles collected at Playa Kalki (12°22′43″N; 69°09′00″W) on 20 September 2008 and maintained in 0.45 µm-filtered seawater in 2 L polystyrene containers. To accurately time the field experiments to the onset of larval settlement (i.e., when larvae first attached to the bottom and started calcification), a subset of larvae were reared in polystyrene Petri dishes (16 replicate Petri dishes, 40 larvae per replicate, see [13] for further details). The choice chamber trials were started on the day these larvae reached competency to settle (29 September 2008) and continued for three days. Over this time period, settlement rate in the laboratory cultures continued to increase and survivorship remained unchanged.

For the choice chamber trials, three submersible speakers (details below) were arranged in a triangular pattern, and two transparent PLEXIGLAS® chambers (1 m length, 10 cm Ø) were placed in front of each speaker with the near end of the chamber at a distance of 1 m (Figure 1). We introduced ∼500 larvae to each chamber and secured both ends with 50 µm nylon mesh. The distribution of larvae within the chambers was observed *in situ* on three consecutive nights using flashlights between 0400–0500 h.

### Sound experiments

**Figure 3:**
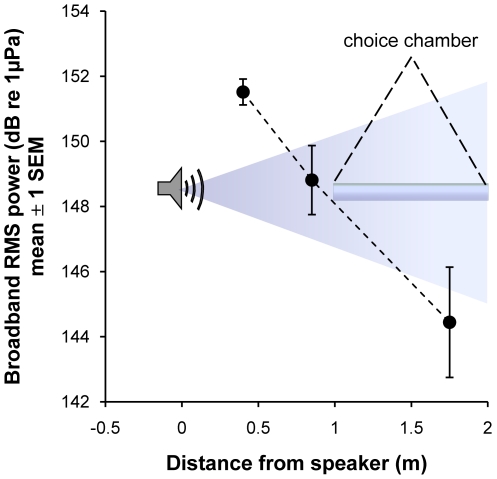
The experimental underwater sound field. Analysis of RMS power gradients on all three axes of the experimental set-up (see Figure 1) during playback showed a 4.4 dB gradient within the chamber. Recordings were taken at three locations along the apparatus. The gradient in the measurements is near to a cylindrical model of geometric spreading (R_L_ = S_L_ – 10 log (R/R_ref_)), as expected for shallow water environments, except that instead of a geometric model parameter of 10, the measured value was 11.1 (SEM = 1.4).

To determine whether coral larvae exhibited a response to general reef noise (rather than to a specific source of reef noise), we broadcast a compilation of recordings of coral reef sounds, which incorporated variation in reef noise due to time-of-day, season, habitat, and depth. Reef sounds were recorded using an omnidirectional hydrophone (HiTech HTI-96-MIN with inbuilt preamplifier, High Tech Inc., Gulfport MS) and an Edirol R-1 24-Bit recorder (44.1 kHz sampling rate, Roland Systems Group, Bellingham WA). Recorded sounds were played back using Electrovoice UW-30 underwater speakers (output level 153 dB re 1 µPa at 1 m, frequency response 0.1 to 10 kHz, Lubell Labs Inc., Columbus OH), and were broadcast from the 3 speakers in synchrony. The broadcast sound consisted of 15 different 3-minute recordings to avoid potential pseudoreplication introduced by using a single recording in playback experiments [17], creating a 45-minute-loop which was played continuously throughout the night. The recordings consisted of pops and grunts made by fishes, background crackling sounds produced by snapping shrimp and occasional sounds of animals feeding, moving, and calling. These sounds were recorded at a variety of different locations (Piscadera, Spaanse Water), habitats (reef plateau at 5 m, reef slope at 15 m), dates (August 2006, March 2007) and lunar phases (over a 20 day period in March 2007), and times of day and night (between 0715 and 2200 h). To determine whether there was a measurable gradient in sound in the chamber, we took recordings during playback at three locations along the axis of each chamber, and used Avisoft-SASLab Pro (Avisoft Bioacoustics, Berlin, Germany) to calculate the mean RMS (Root Mean Square) broadband intensity at each location. We used recordings of a pure tone 1000 Hz sine wave produced by a signal generator (TTi TG230, Thurlby Thandar Instruments, Huntingdon UK) and the manufacturer's calibration of the hydrophone to calibrate the recordings to dB re 1 µPa (Figure 3). Acoustic pressure was 148.9 dB re 1 µPa at the near end compared to 144.5 dB re 1 µPa at the far end of the chamber, demonstrating a clear gradient in pressure level through the chamber, and implying that geometric spreading from the speakers was approximately cylindrical in nature: RL = SL – 11.1 log (R/R_ref_). Using p = ρ*c*v (where p =  pressure in Pa, ρ =  water density in kg m^−3^, *c* =  speed of sound in m s^−1^, and v =  particle velocity in m s^−1^), the gradient in particle velocity in the choice chamber was from 9.64×10^−8^ m s^−1^ at the near end to 9.35×10^−8^ m s^−1^ at the far end.

## References

[1] Gleason DF, Danilowicz BS, Nolan CJ (2009). Reef waters stimulate substratum exploration in planulae from brooding Caribbean corals.. Coral Reefs.

[2] Birrell CL, McCook LJ, Willis BL, Diaz-Pulido GA (2008). Effects of benthic algae on the replenishment of corals and the implications for the resilience of coral reefs.. Oceanogr Mar Biol Ann Rev.

[3] Armsworth PR (2000). Modelling the swimming response of late stage larval reef fish to different stimuli.. Mar Ecol Prog Ser.

[4] Montgomery JC, Jeffs A, Simpson SD, Meekan MG, Tindle C (2006). Sound as an orientation clue for the pelagic larvae of reef fish and crustaceans.. Adv Mar Biol.

[5] Kingsford MJ, Leis JM, Shanks A, Lindeman KC, Morgan SG (2002). Sensory environments, larval abilities and local self-recruitment.. Bull Mar Sci.

[6] Simpson SD, Meekan MG, McCauley RD, Jeffs A (2004). Attraction of settlement-stage coral reefs fishes to ambient reef noise.. Mar Ecol Prog Ser.

[7] Simpson SD, Meekan MG, Montgomery JC, McCauley RD, Jeffs A (2005). Homeward sound.. Science.

[8] Kernan MJ (2007). Mechanotransduction and auditory transduction in *Drosophila*.. Pflugers Arch.

[9] Robert D (2009). Insect bioacoustics: mosquitoes make an effort to listen to each other.. Curr Biol.

[10] Mann DA, Cott PA, Hanna BW, Popper AN (2007). Hearing in eight species of northern Canadian freshwater fishes.. J Fish Biol.

[11] McCauley RD (1997). Aspects of marine biological sounds in northern Australia IV: Fish choruses in the Great Barrier Reef, spatial extent and temporal patterns..

[12] Cato DH (1992). The biological contribution to the ambient noise in waters near Australia.. Acoustics Aus.

[13] Vermeij MJA, Fogarty ND, Miller MW (2006). Variable planktonic conditions affect larval behavior, survival, and settlement patterns in the Caribbean coral *Montastraea faveolata*.. Mar Ecol Prog Ser.

[14] Morse DE, Hooker N, Morse ANC, Jensen RA (1988). Control of larval metamorphosis and recruitment in sympatric agariciid corals.. J Exp Mar Biol Ecol.

[15] Mann DA, Casper BM, Boyle KS, Tricas TC (2007). On the attraction of larval fishes to reef sounds.. Mar Ecol Prog Ser.

[16] Swearer SE, Shima JS, Hellberg ME, Thorrold SR, Jones GP (2002). Evidence of self-recruitment in demersal marine populations.. Bull Mar Sc.

[17] Slabbekoorn H, Bouton N (2008). Soundscape orientation: a new field in need of sound investigation.. Anim Behav.

